# CatLC: Catalonia Multiresolution Land Cover Dataset

**DOI:** 10.1038/s41597-022-01674-y

**Published:** 2022-09-08

**Authors:** Carlos García, Oscar Mora, Fernando Pérez-Aragüés, Jordi Vitrià

**Affiliations:** 1 Institut Cartogràfic i Geològic de Catalunya, Barcelona, 08038 Spain; 2 Universitat de Barcelona, Departament de Matemàtiques i Informàtica, Barcelona, 08007 Spain

**Keywords:** Natural hazards, Environmental impact

## Abstract

The availability of large annotated image datasets represented one of the tipping points in the progress of object recognition in the realm of natural images, but other important visual spaces are still lacking this asset. In the case of remote sensing, only a few richly annotated datasets covering small areas are available. In this paper, we present the Catalonia Multiresolution Land Cover Dataset (CatLC), a remote sensing dataset corresponding to a mid-size geographical area which has been carefully annotated with a large variety of land cover classes. The dataset includes pre-processed images from the Cartographic and Geological Institute of Catalonia (ICGC) (https://www.icgc.cat/en/Downloads) and the European Space Agency (ESA) (https://scihub.copernicus.eu) catalogs, captured from both aircraft and satellites. Detailed topographic layers inferred from other sensors are also included. CatLC is a multiresolution, multimodal, multitemporal dataset, that can be readily used by the machine learning community to explore new classification techniques for land cover mapping in different scenarios such as area estimation in forest inventories, hydrologic studies involving microclimatic variables or geologic hazards identification and assessment. Moreover, remote sensing data present some specific characteristics that are not shared by natural images and that have been seldom explored. In this vein, CatLC dataset aims to engage with computer vision experts interested in remote sensing and also stimulate new research and development in the field of machine learning.

## Abstract (summary)

**Table:** 

Measurement(s)	RGB orthophoto image • Infrared orthophoto image • Radar backscattering • Hyperspectral image • Topographic measurements • Landcover classification
Technology Type(s)	Airborne camera • Satellite SAR sensor • Satellite hyperspectral sensor • LiDAR and optical airborne sensors • Optical and hyperspectral sensors

## Background & Summary

Pixel-wise classification of remote sensing images is a challenging task that often requires fieldwork and manual annotation because of the importance of its role in several critical applications. Therefore, mapping agencies and organizations are on the quest to explore how to minimize these strenuous and time-consuming manual tasks by using computer-assisted processes. To do so, current automatic land cover segmentation techniques have benefited from better raw data sources, but they still require improvement in terms of accuracy and also better integration strategies with humans in the loop.

Land cover mapping is among the primary use cases of airborne and satellite images and the proposed focus of this work. Land cover maps are used in different applications such as forest inventory and management, hydrology, crop management or geologic risk identification and assessment. Therefore, accurate and updated knowledge about land dynamics is essential for territory management with different purposes and in multiple fields but, nowadays, high-resolution land segmentation is still done mainly by employing photointerpretation techniques, entailing high costs in terms of time and human resources.

The transformation towards a computer-assisted solution faces a critical point: the scarcity of high-quality datasets, being the labeling process one of the main causes of this situation. Labeling natural image datasets, such as ImageNet^1^ and PASCAL VOC^2^, does not pose an interpretation problem as their classes are well defined, distinctive, and can be easily understood by any human annotator. However, labeling remote sensing images correctly might need expert knowledge and requires access to different sources of raw data. For example, differentiating between deciduous or evergreen forest is not an easy task even for the expert.

When data are scarce, the best strategy when developing classification models is to adapt models that have been developed in similar fields with plenty of data. The case of remote sensing is not an exception, and the use of traditional natural image segmentation architectures is the paradigm in the field. There are some reasons to think that these models are not optimal for remote sensing images because of their inadequate inductive biases, but this hypothesis can only be validated by having access to large datasets of carefully labeled remote sensing data. It is necessary to tackle issues such as the restricted translational invariance of these images or the variable resolution of their bands.

Additionally, in the near future, full automation of high-resolution cartographic tasks such as land mapping will be the norm, and better strategies to develop powerful deep learning models with a human in the loop are necessary. Traditional pixel-wise deep learning segmentation techniques must also be adapted to this end^3^.

**Fig. 1:**
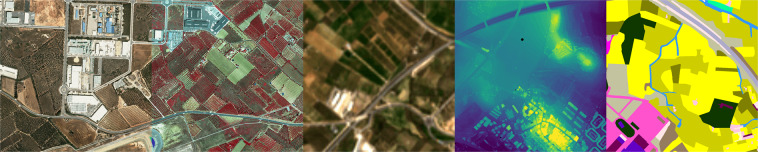
Continuous area using different layers of the dataset together with the ground truth labels.

In this paper, we present the Catalonia Multiresolution Land Cover Dataset (CatLC). This dataset (see Fig. 1) comprises a large variety of images: RGB and infrared orthophotos from airborne sensors at high resolution, radar imagery from Sentinel-1 satellites, multispectral data from Sentinel-2 satellites and compositions of topographic maps–all those accompanied by a land cover map labeled by experts in photointerpretation. Using different combinations of images from the dataset, we offer a benchmark that could serve as a starting point to explore different artificial intelligence techniques for remote sensing segmentation purposes. CatLC dataset aims to engage with computer vision experts interested in remote sensing and stimulate research and development.

## Methods

In this section, the CatLC dataset is presented in detail. It includes a set of images, obtained by airborne and satellite sensors, from the catalogs of the Cartographic and Geological Institute of Catalonia (ICGC) and the European Space Agency (ESA). Labels correspond to the current ICGC’s land cover map.

**Fig. 2:**
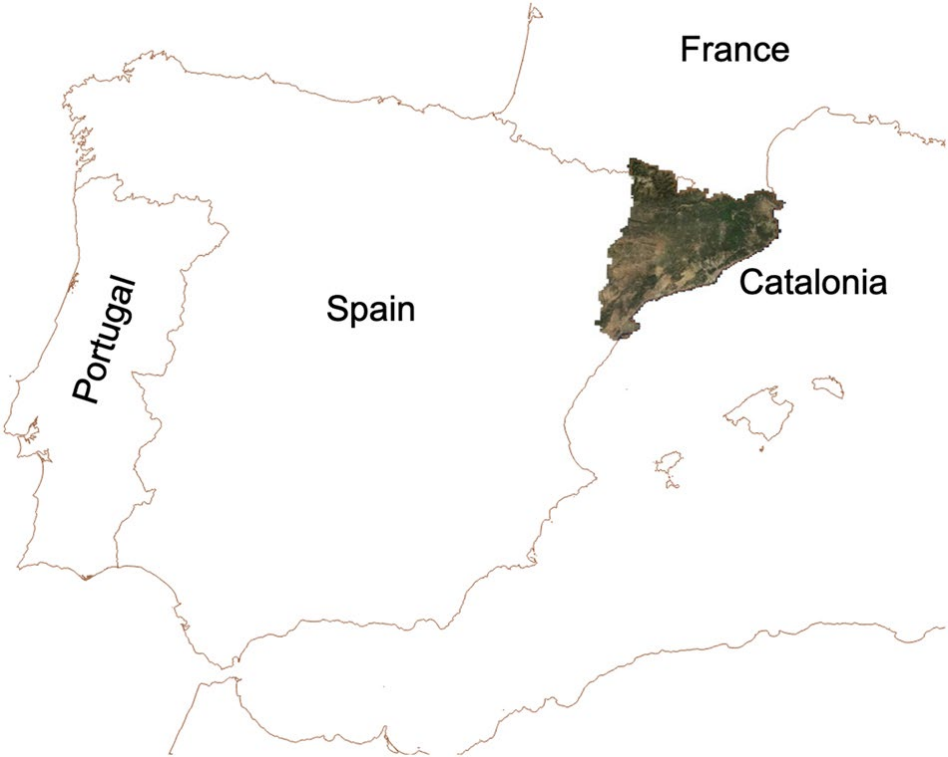
Location of the area of interest, Catalonia (Spain).

The CatLC dataset covers the entire territory of Catalonia (Spain) (see Fig. 2), approximately 32000 *km*^2^, providing a high quality source of information for the application of Artificial Intelligence (AI) and Deep Learning (DL) techniques, both regarding the variety of the information and their extension.

All the images were acquired during 2018 at different spectral bands and spatial resolutions. They are provided in GeoTiff raster format and share a common georeferencing system projection (WGS84 UTM31N Reference System). They cover the same geographic extension, given by the following bounding box: UTM X West: 240000, UTM X East: 540000, UTM Y North: 4780000, and UTM Y South: 4480000.

The different data layers, with spatial resolutions varying between 1 and 10 meters depending on the product and sensor used, are presented in detail in the following subsections. In such subsections we illustrate the images provided by the dataset (land cover, orthophoto, Sentinel-1, Sentinel-2 and topographic maps). Figures 5, 6, 7, 8, 9, 11, 12 and 13 show different images on three geographical areas in Catalonia. We have summarized all available data in Table 1.

**Table 1:** Summary of CatLC dataset.

Data	Independent Bands	Resolution (m)
Orthophoto RGB	4	1
Sentinel-1	12	10
Sentinel-2	20	10
DEM	4	5
DSM	4	1
CHM	4	1
Land cover map	—	1

34040 images at 960 × 960 pixels per source of 1 m data.

### Land cover map

**Fig. 3:**
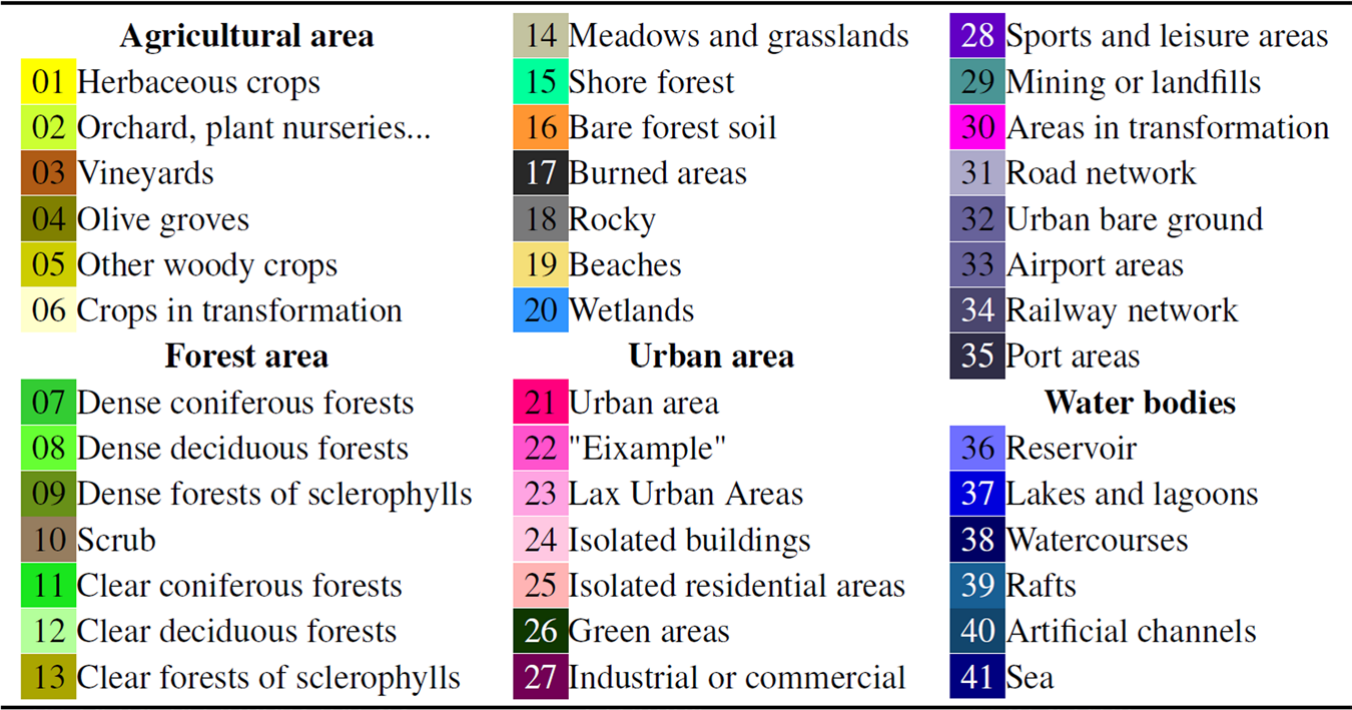
CatLC dataset with 41 classes and legend.

The 2018 land cover map presented here has 41 different classes (see Fig. 3), including different agricultural areas, forest areas, urban areas and water bodies. Photointerpreters from ICGC followed a standardized procedure during its generation process. The minimum area for labeling an element was 500 squared meters and the minimum length for linear features such as roads, rivers, railroad tracks, etc. was between 8 and 10 meters (https://datacloud.ide.cat/especificacions/cobertes-sol-v1r0-esp-01ca-20160919.pdf).

Supervision has been performed on a sample of 811 points throughout this territory, resulting in a thematic accuracy of 81%. The final 41 labels (see Fig. 3) presented in this publication are delivered at spatial resolution of 1 m.

**Fig. 4:**
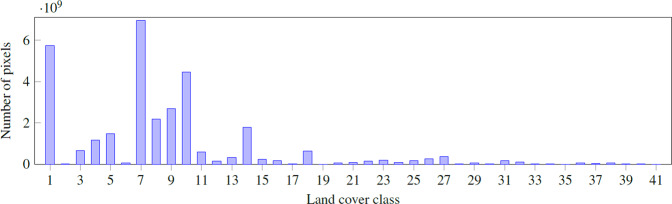
Class distribution on CatLC dataset.

**Fig. 5:**
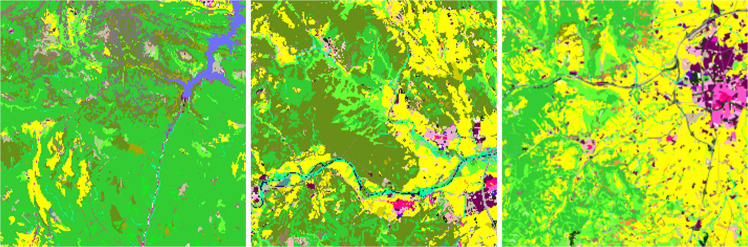
The distribution of the land covers within the mapped territory is heterogeneous. Some covers as herbaceous crops or dense coniferous forests are much more common than airport areas or water bodies. In Fig. 4, we can see the histogram for the complete dataset.

The distribution of the land covers within the mapped territory is heterogeneous. Some covers as herbaceous crops or dense coniferous forests are much more common than airport areas or water bodies. In Fig. 4 we can see the histogram for the complete dataset (see also Fig. 5).

### Orthophoto

An orthophoto is a cartographic document consisting of a vertical aerial image that has been rectified in such a way as to maintain a uniform scale over the entire image surface. It consists of a geometric representation at a given scale of the Earth’s surface.

Original images were taken with a resolution of 25 centimeters, but because the land cover map has a resolution of 1 meter, we have decided to rescale the orthophoto raster layer also to 1 meter.

**Fig. 6:**
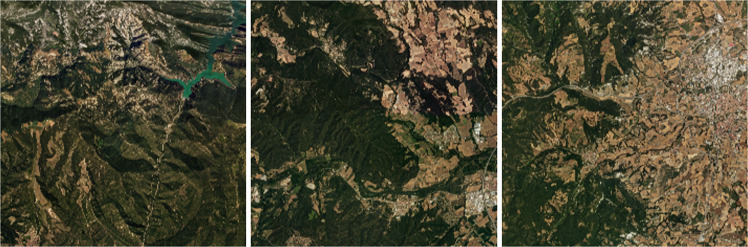
Orthophoto RGB samples.

**Fig. 7:**
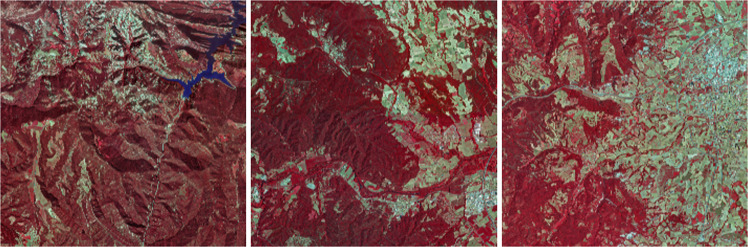
Orthophoto (Infrared,R,G) samples.

This layer comprises four distinct bands, each providing information from different zones of the electromagnetic spectrum. Three of them belong to the visible area of the spectrum (RGB) (see Fig. 6) and one of them to the infrared area (see Fig. 7). A continuous image is generated based on several thousands of independent photoshoots processed with a combination of commercial software (Trimble/Inpho) and in-house developments. On this cartographic document, digital retouching tasks have been carried out to minimize artifacts that may have originated during the acquisition and processing of the images. The applicable specification can be found in^4^.

### Sentinel-1

**Fig. 8:**
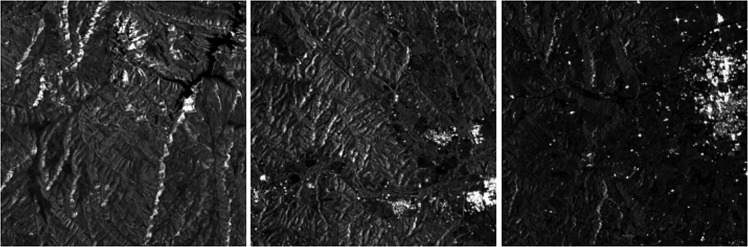
Sentinel-1 (average image during 2018) samples.

The Sentinel-1 dataset has been generated from Synthetic Aperture Radar (SAR) images in GRD (Ground Range Detected) mode from the year 2018 at 10-meter spatial resolution. The Sentinel-1 satellite constellation is made up of two twin satellites, A and B, from the European Space Agency (ESA). These satellites emit a microwave signal (frequency 5.405 GHz) and subsequently receive the echo of the reflection on the ground surface. Therefore, Sentinel-1 images contain information on the reflectivity of the terrain that depending on its type (urban, vegetation, crops, water, etc.) will have different intensities, thus providing valuable information for land cover classification. For this purpose, 12 acquisitions have been chosen, one for each month of the year, covering the entire territory of Catalonia. Full coverage has been achieved by combining two orbits in ascending mode (orbits 30 and 132) and VV (Vertical-Vertical) polarizations in similar dates. The descending orbit and VH (Vertical-Horizontal) polarization have not been included in the present dataset because the information is mostly redundant. However, its use can be explored in case it provides improvements in segmentation. Additionally, an average image of the year 2018 has been generated with improved radiometry (multitemporal speckle reduction) by combining all 12 monthly images into one (see Fig. 8). Consequently, the average image cannot provide information on temporary changes during 2018 but does provide a lower noise-level image.

The images were processed with the SNAP (Sentinel Application Platform) software^5^ from ESA using the following procedure:

1. Download of the precise orbit for each image using the “Apply-Orbit-File” function, which provides detailed information for its correct georeferencing.
2. Deletion of noisy pixels from the edge of the image using the “Remove-GRD-Border-Noise” function.
3. Radiometric calibration of each image providing calibrated reflectivity information for the Sentinel-1 images. A correct calibration is necessary for the multitemporal study of the data.
4. Topographic effects Compensation using the “Terrain-Flattening” function. The acquisition geometry of the SAR images is oblique, which generates distorting artifacts in the reflectivity associated with the terrain topography (layover, foreshortening and shadowing). This processing compensates for these artifacts to obtain an image that is as independent as possible from the topography.
5. Georeferencing using the “Terrain-Correction” function and final mosaicking of the images.

A video comparing the average Sentinel-1 image and the image for each month is on the CatLC webpage^6^.

### Sentinel-2

**Fig. 9:**
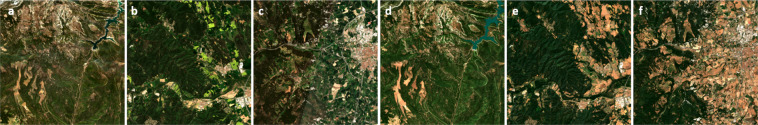
Sentinel-2 RGB April (**a–c**) and August (**d–f**) 2018 samples.

Sentinel-2 provides multispectral imagery data at different resolutions approximately every five days. We have selected two relevant dates for this dataset, the first one in April and the second one in August. We have chosen these two dates to follow the phenological evolution of the vegetation throughout the spring and late summer. As we are in the Mediterranean area, with these two dates it is possible to detect both winter and summer herbaceous crops as well as evergreen and deciduous forest areas. Due to the presence of clouds, multiple data takes have been necessary to make a cloud-free mosaic (see Fig. 9).

The images obtained by the MSI sensor from the Sentinel-2A and 2B satellites, from the European Commission Copernicus program, have been atmospherically corrected by means of the ESA sen2cor v2.8 software^7^ to yield Level-2A images.

**Fig. 10:**
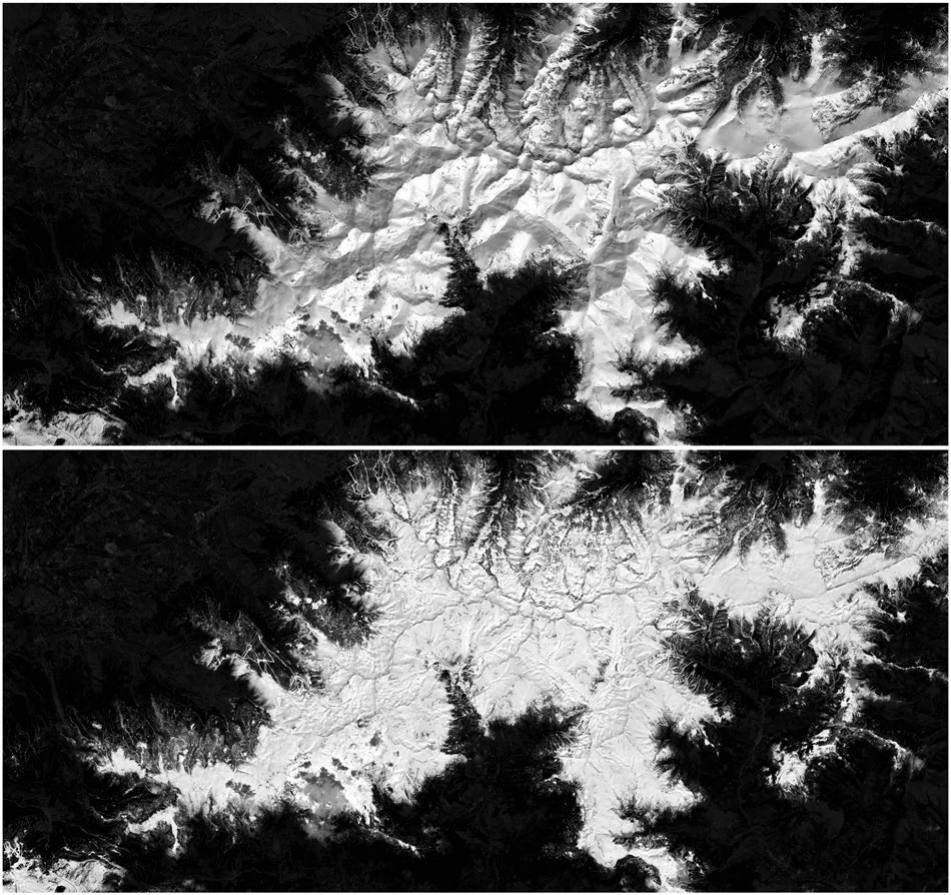
Sentinel-2 process with atmospheric and topographic corrections. Original (up) and corrected image (down).

The main purpose of sen2cor is to correct single-date Sentinel-2 Level-1C Top-Of-Atmosphere (TOA) radiance from the effects of the atmosphere in order to deliver a Level-2A Bottom-Of-Atmosphere (BOA) reflectance. The process may optionally use a DEM (Digital Elevation Model) to correct the changes in the radiometry related to the topographic relief. A 10 m gridded DEM generated at ICGC by photogrammetric techniques has been used in this study. A total of 10 bands, at 10 m and 20 m resolution, are preserved as input features for the Deep Learning process. Figure 10 presents Sentinel-2 images before and after they have been corrected.

### Topographic layers

Three different topographic products and two subproducts were generated. Their characteristics are outlined in the following sections.

#### Digital elevation model

**Fig. 11:**
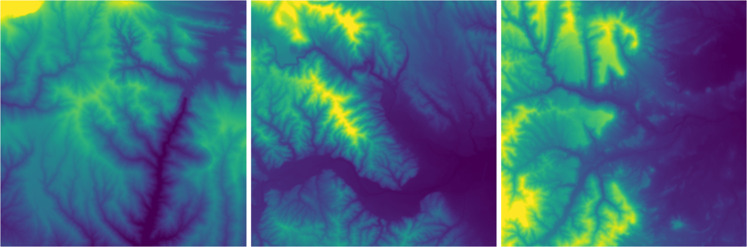
Digital Elevation Model (DEM) samples.

This is a standard layer freely distributed by ICGC and is built upon the altimetric information of the Topographic Base of Catalonia 1:5000 version 2 (BT-5m v2.0) that includes profiles, altimetric coordinates, break and contour lines, all of them obtained from the terrain. It consists of a raster image at 5 m pixel size and its estimated altimetric accuracy is 0.9 m RMS (see Fig. 11). The specification followed for its generation can be found in^8^.

Two typical subproducts for remote sensing applications are the slope, which indicates each pixel’s steepness, and the aspect, yielding the orientation of the maximum slope between adjacent pixels. These values have been calculated from the DEM and thus contain redundant information. We include them because they might be helpful for the interpretation of the results.

#### Digital surface model

**Fig. 12:**
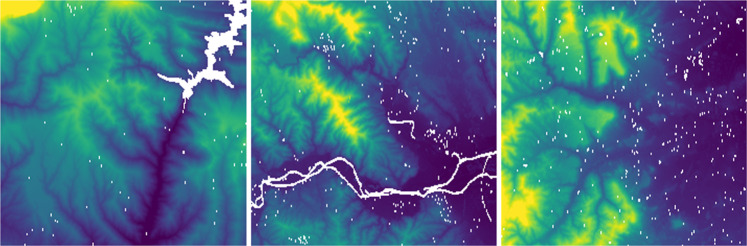
Digital Surface Model (DSM) samples.

The Digital Surface Model (DSM) is a raster layer at 1 m pixel size containing orthometric heights. It represents the topmost height for every pixel position on the grid, be it the ground or features such as forest canopy and buildings (see Fig. 12).

It is generated using Trimble/Inpho’s software package MATCH-T DSM. It works fully automatically using different image matching techniques like feature-based matching (FBM), cost-based matching (CBM) and least squares matching (LSM) to produce highly dense point clouds. The process follows a hierarchical approach starting from an upper level of the image pyramid and generating an approximate DSM for the next lower pyramid level. Different levels of smoothing can be applied as a function of terrain roughness to filter or reject outliers from the generated point cloud. Large point clouds (>5 mio. points) are automatically split into a squared tile structure. From the final point cloud (tiles) a raster file with the selected 1-m grid size is interpolated. The same aerial photogrammetric images at 0.25 m–0.35 m used to produce the orthophoto are employed, thus guaranteeing a good consistency between these products.

#### Canopy height model

**Fig. 13:**
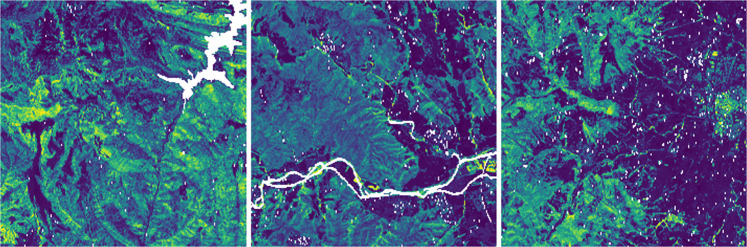
Canopy Height Model (CHM) samples.

The Canopy Height Model (CHM) is a high resolution (1 m) raster dataset that maps all the objects over the terrain as a continuous surface. It is advantageous to delineate the forest extent, but it also includes urban landscape data. Each pixel of this model represents the height of the trees above the ground topography. In urban areas, the CHM represents the height of buildings or other built objects (see Fig. 13).

This layer is created through subtraction of the 2016–2017 LiDAR DEM (interpolated from the standard 2 m-pixel ICGC standard product^9^) from the 2018 photogrammetric DSM. Note that this product is not dependent on the aforementioned Digital Elevation Model.

## Data Records

The complete CatLC dataset is available at the following link^6^. The data files and their formats are detailed in Table 2.

**Table 2:** Detailed description of the different files in the dataset.

Data	Type	Dimensions (pixels)	Pixel Resolution	Size
Orthophoto RGB	GeoTiff	300.000 × 300.000	1 m	252,3 GB
Orthophoto IR	GeoTiff	300.000 × 300.000	1 m	84,1 GB
Sentinel-1 January	GeoTiff	30.000 × 30.000	10 m	3,4 GB
Sentinel-1 February	GeoTiff	30.000 × 30.000	10 m	3,4 GB
Sentinel-1 March	GeoTiff	30.000 × 30.000	10 m	3,4 GB
Sentinel-1 April	GeoTiff	30.000 × 30.000	10 m	3,4 GB
Sentinel-1 May	GeoTiff	30.000 × 30.000	10 m	3,4 GB
Sentinel-1 June	GeoTiff	30.000 × 30000	10 m	3,4 GB
Sentinel-1 July	GeoTiff	30.000 × 30.000	10 m	3,4 GB
Sentinel-1 August	GeoTiff	30.000 × 30.000	10 m	3,4 GB
Sentinel-1 September	GeoTiff	30.000 × 30.000	10 m	3,4 GB
Sentinel-1 October	GeoTiff	30.000 × 30.000	10 m	3,4 GB
Sentinel-1 November	GeoTiff	30.000 × 30.000	10 m	3,4 GB
Sentinel-1 December	GeoTiff	30.000 × 30.000	10 m	3,4 GB
Sentinel-1 Mean 2018	GeoTiff	30.000 × 30.000	10 m	3,4 GB
Sentinel-2 April	GeoTiff	30.000 × 30.000	10 m	16,8 GB
Sentinel-2 August	GeoTiff	30.000 × 30.000	10 m	16,8 GB
Topography - Aspect	GeoTiff	60.000 × 60.000	5 m	13,4 GB
Topography - CHM	GeoTiff	300.000 × 300.000	1 m	336,4 GB
Topography - DEM	GeoTiff	60.000 × 60.000	5 m	13,4 GB
Topography - DSM	GeoTiff	300.000 × 300.000	1 m	336,4 GB
Topography - Slope	GeoTiff	60.000 × 60.000	5 m	13,4 GB
Land Cover Map	GeoTiff	300.000 × 300.000	1 m	84,1 GB

## Technical Validation

Assessing the quality of the images in the dataset is very important to ensure that the input data is of optimal quality, apart from its characteristics such as the spatial resolution or the number of spectral bands. In order to show the quality of the presented data, bellow there is a summary of the Quality Controls (QC) used during their processing:

1. Land Cover map: The land cover map update process includes periodic checks of some selected polygons among those that have been geometrically and semantically modified. In the case of systematic errors or misunderstandings about the legend, they are corrected. At the end of the 2018 update, an internal quality supervision has been carried out on a sample of 811 points throughout the territory, resulting in a thematic accuracy of 81%.
2. Orthophoto: According to the specification^4^, several automatic and manual checks are carried out. The main ones are positional accuracy (RMSE 0.5 m), geometric and radiometric continuity, dynamic range and image quality. Additional manual inspection after retouch ensures that remaining artifacts cover less than 1% of the total area of Catalonia.
3. Sentinel-1 and Sentinel-2: The processing of the Sentinel-1 and Sentinel-2 images has been carried out with the quality standards of the ESA SNAP^5^ and sen2cor^7^ software respectively. This guarantees a good radiometric and geometric calibration of the images. After processing, an evaluation of the location of various control points has been performed to validate a geolocation error of less than one pixel (10 m) in absolute value and relative to the rest of the products in the dataset
4. Digital Elevation Model: As stated in^8^ the resulting DEM is checked for positional accuracy (RMSE 0.9 m), logical consistency and completeness.
5. Digital Surface Model and Canopy Height Model: The quality of both the LiDAR Digital Terrain Model (DTM) and the DSM was checked against a high number of independent check points, measured with GPS at a vertical accuracy of approx. 4 cm. In case of the LiDAR DTM, the check points are located on soccer fields. For the DSM the available country-wide network of photogrammetry control points was used. Only points located on the ground were selected (around one thousand points). From the check points the following empirical vertical accuracy values (Root Mean Squared Errors - RMSE) were derived:
  LiDAR DTM: better than 15 cm.
  DSM in the Pyrenees: better than 40 cm.
  DSM in the rest of Catalonia: better than 30 cm.
  CHM in the Pyrenees: better than 45 cm.
  CHM in the rest of Catalonia: better than 35 cm.

Since the DSM and the CHM are automatically generated products, their quality can be considerably decreased in areas where the matching algorithm did not achieve optimal results (e.g. in shadow areas).

It should be also noted that in areas covered with some kind of forests and mildly sparse trees the DSM/CHM does not always represent the height of the canopy, depending on the tree density and the presence of foliage.

## Usage Notes

An initial benchmark accompanies the CatLC dataset as a starting point and to show a helpful pipeline to train a model with provided data. Note that the use of these data is subject to a Creative Common International Recognition 4.0 license, and contains Sentinel Copernicus data modified by the ICGC.

**Fig. 14:**
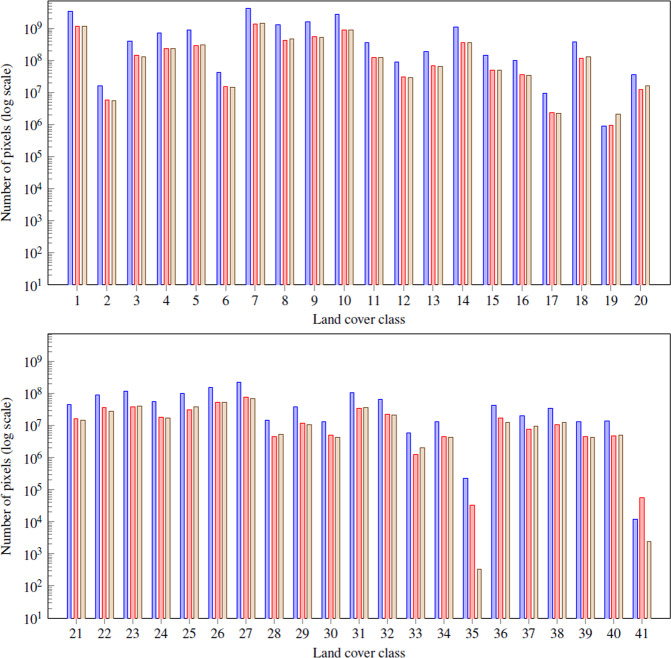
Distribution of the CatLC dataset in three sets: Blue for the train set, red for the validation set and brown for the test set.

Unlike other datasets that have multiple images, CatLC has only one large image. To work with it, we will need to access smaller tiles, so the first step has been to create a list with the indexes of all the tiles that we are going to use in the dataset of dimension 960 × 960 pixels (in the higher spatial resolution images of 1 m). This list was then randomly divided into three groups, 60% for training, 20% for validation, and 20% for testing. Being this a segmentation problem, we have not been able to have a homogeneous distribution of the three groups because usually tiles contain multiple classes. The distribution for the sets can be found in Fig. 14.

As the main baseline, we have selected the classical U-Net neural network^10^, which is used as a starting point in most applications that require image segmentation. This baseline has been implemented in PyTorch, running in a workstation with a Nvidia Quadro P5000 GPU. Cross entropy loss has been used, together with an Adam optimizer with 0.0001 as the learning rate.

Experiments consider three different scenarios:

1. Use as input data RGB orthophotos and infrared band. We know by experience that the high resolution should give good results in the border between different classes, but its limitations regarding spectral bands makes it harder to differentiate classes that belong to the agricultural or forest superclasses.
2. Use as input data two Sentinel-2 images corresponding to April and August. This time, the low resolution will penalize the frontiers, but there should be an improvement in differentiating agricultural or forest superclasses.
3. Use as input data the complete CatLC dataset. It does not make sense to use Sentinel-1 or topographical data all alone because most of its information is about elevation or reflectivity. But the combinations of those with orthophotos and Sentinel-2 data should improve the results.

**Fig. 15:**
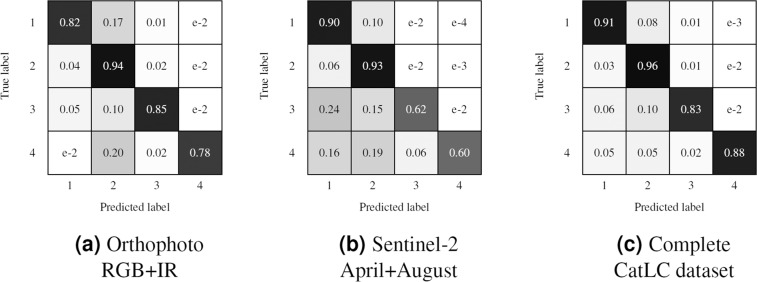
Confusion matrix using different input data. All trained with U-Net neural network. The 41 classes have been compacted to the 4 superclasses (1: agriculture, 2: forest, 3: urban, 4: water).

**Fig. 16:**
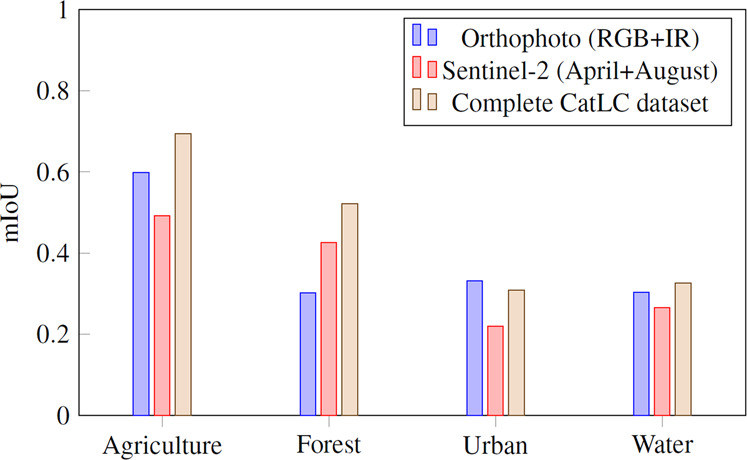
Mean Intersection over Union using different input data for 4 superclasses.

**Fig. 17:**
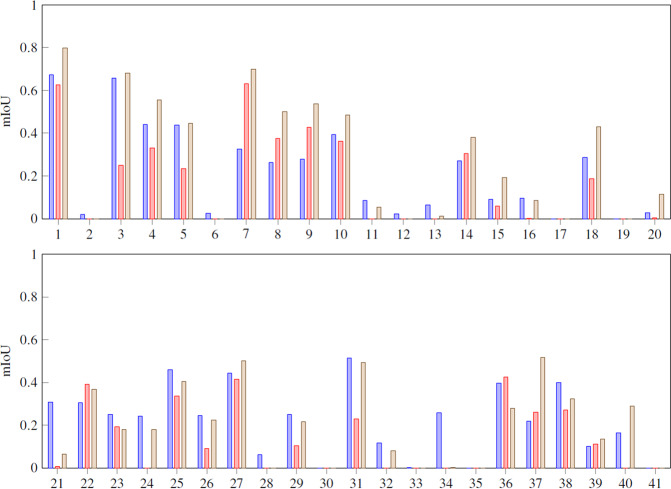
Mean Intersection over Union using different input data for 41 classes.

**Fig. 18:**
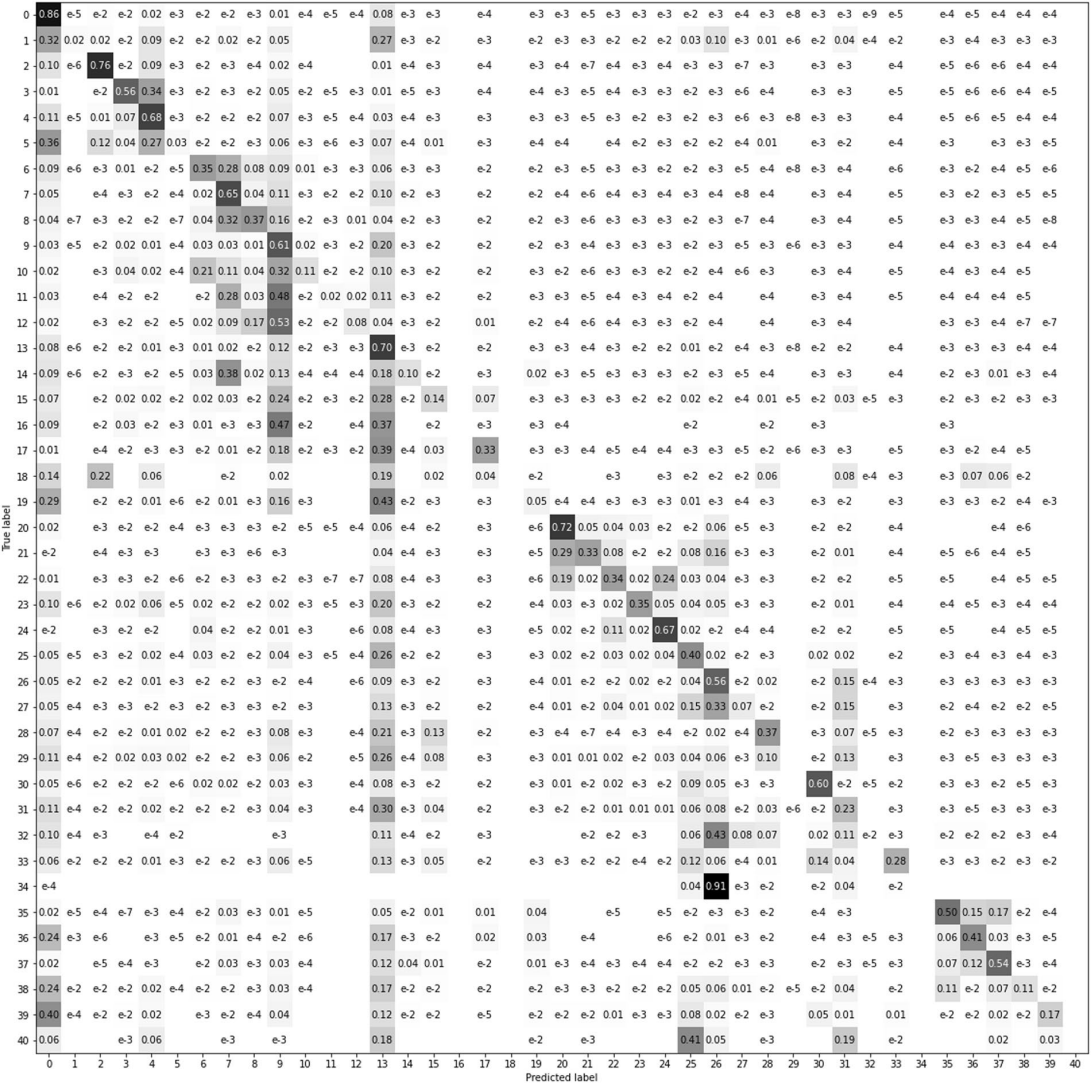
Confusion matrix using the orthophoto (RGB-IR) as input data.

**Fig. 19:**
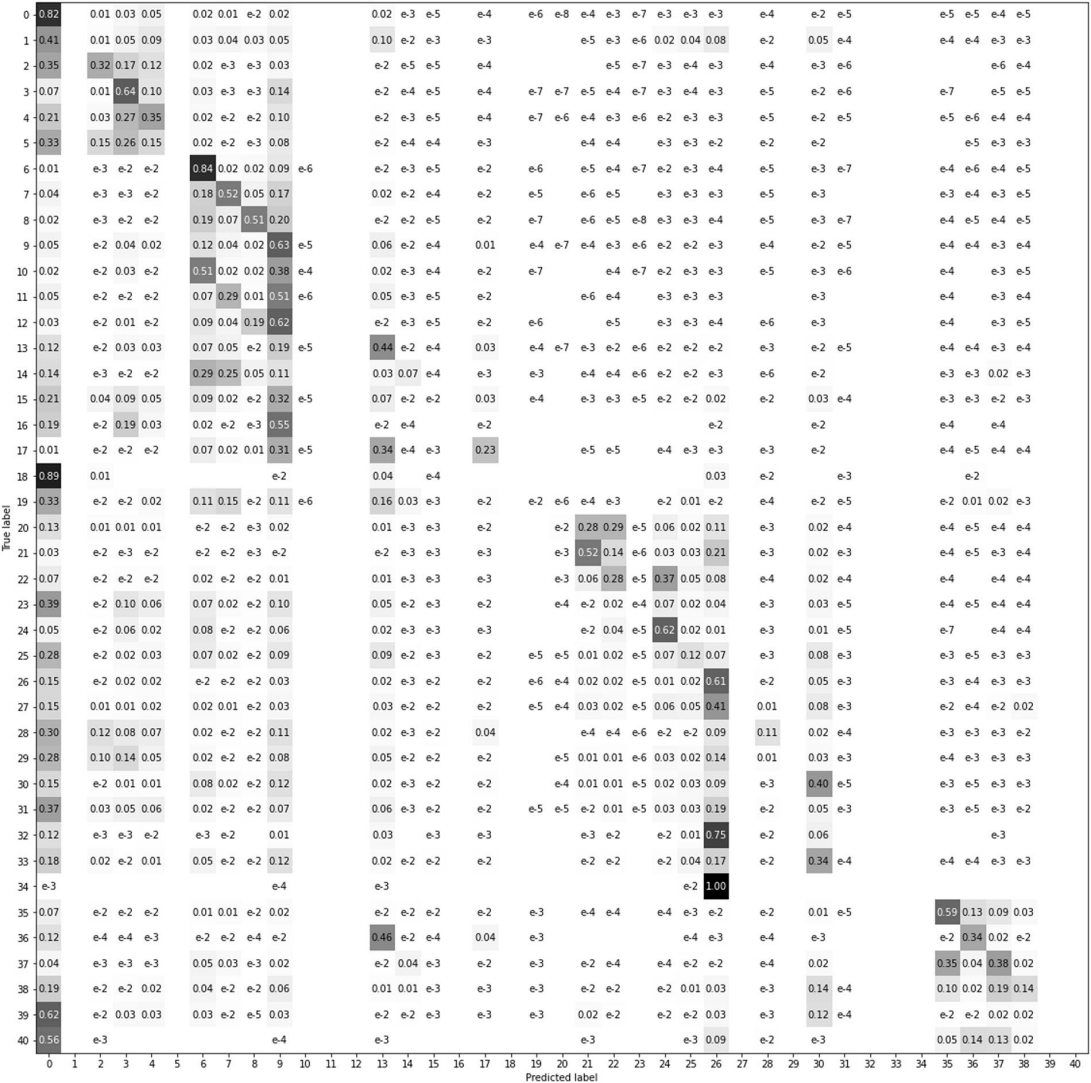
Confusion matrix using Sentinel-2 (April + August) as input data.

**Fig. 20:**
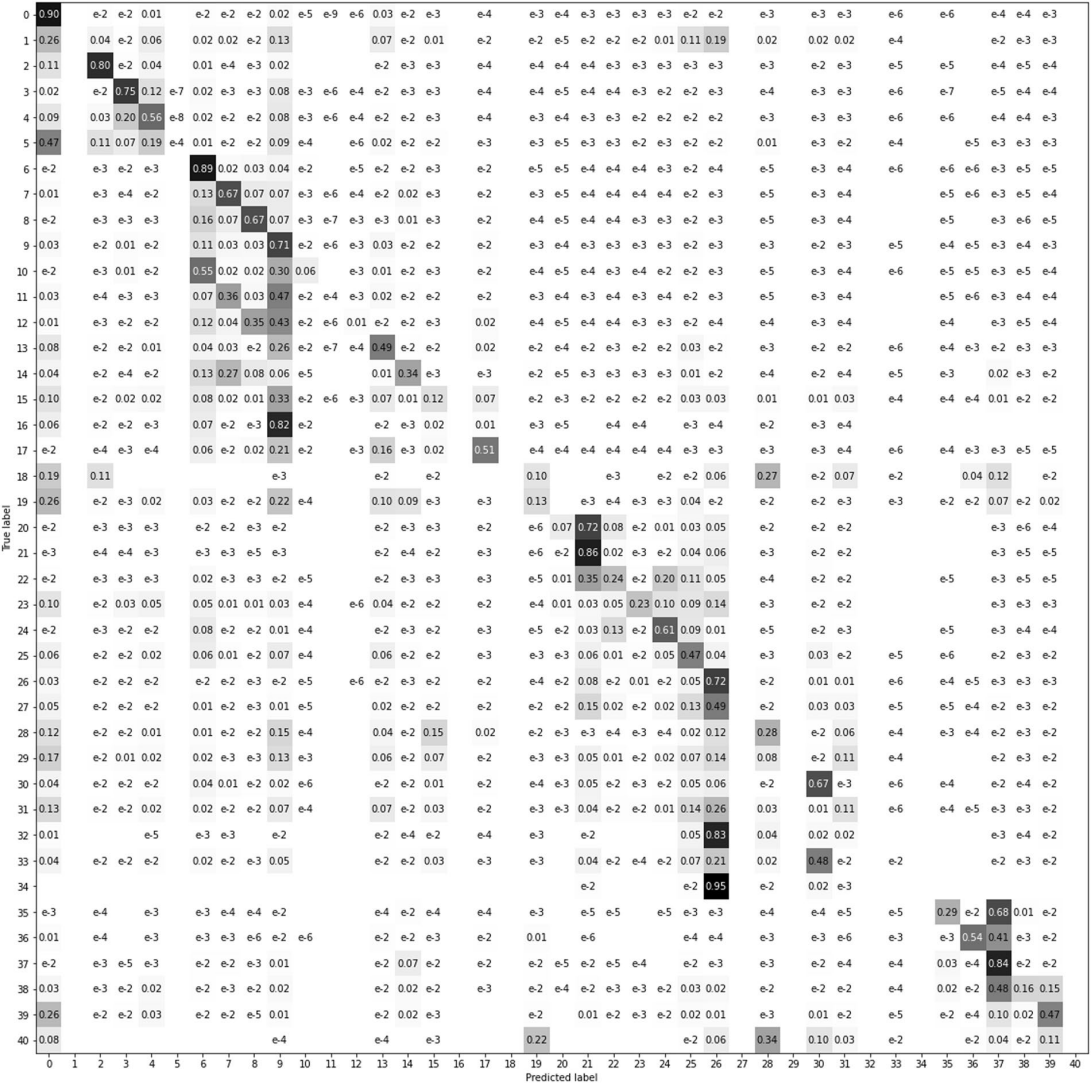
Confusion matrix using the complete CatLC dataset as input data.

To better visualize the results, we have compressed the data in a four superclasses confusion matrix (Fig. 15) and mean intersection over union metrics (Fig. 16) as recommended in COCO dataset^11^. In Figs. 17–20 a confusion matrix and a mean intersection over union for all the 41 classes are shown.

**Fig. 21:**
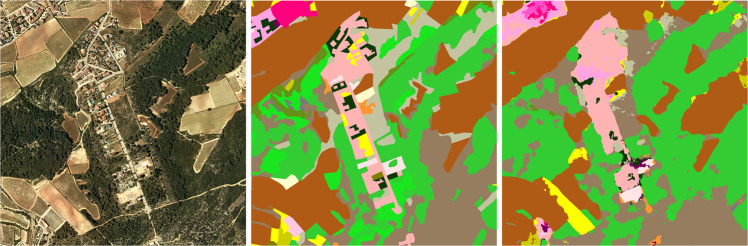
Example of U-Net segmentation: RGB orthophoto, land-cover ground truth, U-Net prediction.

As we stated before, Sentinel-2 outperforms the orthophoto in agricultural and forest zones, but it loses when we need more resolution as in urban areas. Finally, using the complete dataset gives better results overall. In Fig. 21 there is an example of a segmentation using the complete CatLC dataset.

## Data Availability

CatLC is available for download, along with all the necessary information and tutorials, on the following website^6^. There is a tutorial on how to manage the data in the following url: https://github.com/OpenICGC/CatLC/. There is also the code to reproduce the training presented in the article. We provide the logs for the whole training that can be visualized using Tensorboard.
